# Transport characteristics of guanidino compounds at the blood-brain barrier and blood-cerebrospinal fluid barrier: relevance to neural disorders

**DOI:** 10.1186/2045-8118-8-13

**Published:** 2011-02-28

**Authors:** Masanori Tachikawa, Ken-ichi Hosoya

**Affiliations:** 1 Department of Pharmaceutics, Graduate School of Medicine and Pharmaceutical Sciences, University of Toyama, Toyama, Japan

## Abstract

Guanidino compounds (GCs), such as creatine, phosphocreatine, guanidinoacetic acid, creatinine, methylguanidine, guanidinosuccinic acid, γ-guanidinobutyric acid, β-guanidinopropionic acid, guanidinoethane sulfonic acid and α-guanidinoglutaric acid, are present in the mammalian brain. Although creatine and phosphocreatine play important roles in energy homeostasis in the brain, accumulation of GCs may induce epileptic discharges and convulsions. This review focuses on how physiologically important and/or neurotoxic GCs are distributed in the brain under physiological and pathological conditions. Transporters for GCs at the blood-brain barrier (BBB) and the blood-cerebrospinal fluid (CSF) barrier (BCSFB) have emerged as substantial contributors to GCs distribution in the brain. Creatine transporter (CRT/solute carrier (SLC) 6A8) expressed at the BBB regulates creatine concentration in the brain, and represents a major pathway for supply of creatine from the circulating blood to the brain. CRT may be a key factor facilitating blood-to-brain guanidinoacetate transport in patients deficient in *S*-adenosylmethionine:guanidinoacetate *N*-methyltransferase, the creatine biosynthetic enzyme, resulting in cerebral accumulation of guanidinoacetate. CRT, taurine transporter (TauT/SLC6A6) and organic cation transporter (OCT3/SLC22A3) expressed at the BCSFB are involved in guanidinoacetic acid or creatinine efflux transport from CSF. Interestingly, BBB efflux transport of GCs, including guanidinoacetate and creatinine, is negligible, though the BBB has a variety of efflux transport systems for synthetic precursors of GCs, such as amino acids and neurotransmitters. Instead, the BCSFB functions as a major cerebral clearance system for GCs. In conclusion, transport of GCs at the BBB and BCSFB appears to be the key determinant of the cerebral levels of GCs, and changes in the transport characteristics may cause the abnormal distribution of GCs in the brain seen in patients with certain neurological disorders.

## Introduction

**Figure 1:**
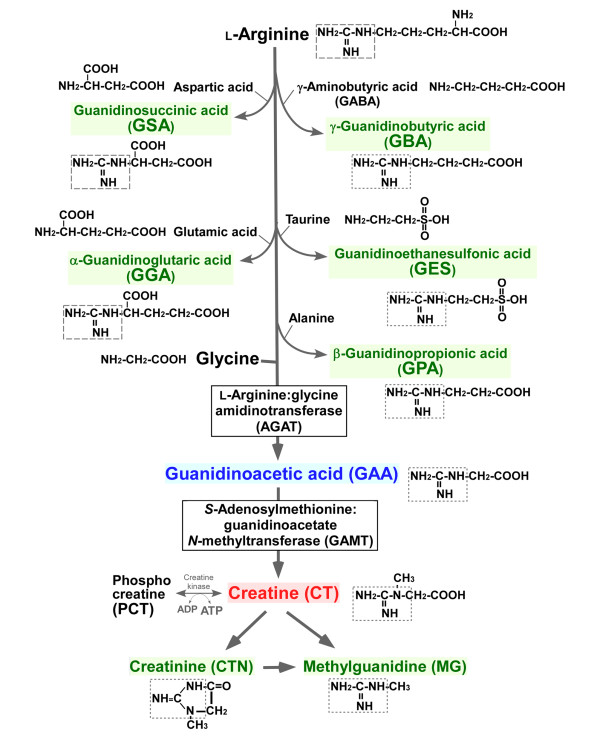
**Putative synthetic pathways of guanidino compounds in the brain**.

Guanidino compounds (GCs), such as creatine (CT), phosphocreatine (PCT), guanidinoacetic acid (GAA), creatinine (CTN), methylguanidine (MG), guanidinosuccinic acid (GSA), γ-guanidinobutyric acid (GBA), β-guanidinopropionic acid (GPA), guanidinoethane sulfonic acid (GES) and α-guanidinoglutaric acid (GGA), are present in the mammalian brain at individual concentrations in the nanomolar to millimolar range [1-3]. CT and PCT play a pivotal role in the storage and utilization of phosphate-bound energy via CT kinase in the brain, thus serving to maintain energy homeostasis in the brain [4]. In this CT/PCT/CT kinase shuttle system, the phosphate-bound energy of ATP is transferred to CT and stored as PCT to be used for regenerating ATP from ADP [4]. On the other hand, GCs are known to be endogenous convulsants [1]. It has been proposed that most GCs are synthesized via transamidination of the amidino group from arginine to amino acids and neurotransmitters [1] (Figure 1). CT biosynthesis involves two sequential steps catalyzed by L-arginine:glycine amidinotransferase (AGAT) and *S*-adenosylmethionine:guanidinoacetate *N*-methyltransferase (GAMT), producing the CT biosynthetic precursor GAA and CT, respectively. CTN is non-enzymatically produced from CT.

The brain barriers might be key determinants of the levels of GCs in the brain and cerebrospinal fluid (CSF). The brain barriers consist of the blood-brain barrier (BBB) and the blood-cerebrospinal fluid barrier (BCSFB). The BBB and BCSFB are formed by tight junctions of brain capillary endothelial cells and choroid plexus epithelial cells, respectively, and serve to regulate the supply of essential nutrients into the brain/CSF and the elimination of xenobiotics and endogenous metabolites from the brain/CSF via various transport systems such as transporters [5,6]. It is thus conceivable that transporters for GCs play an essential role in the influx and efflux transport of GCs at the BBB and/or the BCSFB. However, evidence on the molecular mechanism(s) of GCs transport between the circulating blood and the brain remained incomplete or conflicting. We have investigated the brain barrier transport and cerebral distribution of GCs. In this review, we present an overview of the BBB and the BCSFB transport of GCs and related amino acids and neurotransmitters.

## Relevance of GCs transport at the brain barriers to neural disorders

A physiological importance of CT in the brain has been evidenced in inherited CT deficiency syndrome (CDS), which is characterized by the absence or severe reduction of CT in the brain [7]. Patients exhibit mental retardation, delayed speech and language delay, epilepsy, extrapyramidal signs, and autistic behavior [8]. CDS is associated with genetic defects in AGAT [9,10], GAMT [11], and CT transporter (CRT/solute carrier (SLC) 6A8) [12]. It has been believed that the blood-to-brain CT supply is limited by the BBB, and that cerebral CT is largely derived from biosynthesis in the brain. Indeed, CT has a net positive charge and an estimated log partition coefficient of -2.7 [13], which would not be consistent with diffusion of CT through plasma membranes. Furthermore, oral administration of 20 g CT per day for 4 weeks produced only a 9% increase in total CT in human brain [14]. On the other hand, in patients with AGAT and GAMT deficiency, oral CT administration increased the CT level in the brain [11] and improved the neurologic symptoms [9,15]. However, patients with CRT deficiency do not show improvement of neurological symptoms following oral CT supplementation [16-18], despite the presence of CT biosynthetic enzymes and normal levels of plasma CT. These lines of evidence prompted us to hypothesize that CRT is expressed at the BBB and mediates the transport of CT from the circulating blood to the brain. To test this idea, it is necessary to identify the origin of CT in the brain.

Cerebral accumulation of GAA contributes to neurological complications, such as epilepsy and seizures, in patients deficient in GAMT [8]. It has been postulated that GCs cannot undergo active brain-to-blood efflux across the brain barriers, so that cerebral accumulation of GCs may occur. This is consistent with the concept that one of the functions of the BBB is to retain neurotransmitters and nutrients in the brain. Consequently, increased levels of GCs in the brain may be associated with epileptic discharges and convulsions. GAA is commonly found to be elevated in brains having an epileptogenic focus and in CSF of several animal models of epilepsy [1]. In contrast, it has been reported that endogenous levels of GAA in CSF increased at the onset of convulsions but returned to the basal level after the convulsions had ceased [19], supporting the idea that the brain possesses a clearance system for GCs via the brain barriers. It has recently been established that the brain barriers act as a clearance system for various metabolites and neurotoxic compounds produced in the brain [6]. Clarifying the cerebral clearance system of GCs from the brain and CSF should therefore provide insight into how abnormal accumulation of GCs in the brain and CSF can be prevented.

## Methodology for evaluation of BBB and BCSFB transport

The *in vivo *blood-to-brain and blood-to-CSF influx transports across the BBB and BCSFB have been evaluated by means of integration plot analysis and the brain uptake index (BUI) method after intravenous and carotid artery injection of radiolabeled compounds, respectively [20-22]. This method enables us to calculate the apparent brain/CSF uptake clearance (CL_influx, BBB_, CL_influx, BCSFB_). The *in vivo *brain-to-blood efflux transport clearance (CL_efflux, BBB_) across the BBB has been determined by means of the brain efflux index (BEI) method [23]. After microinjection of a mixture of radiolabeled test and reference compounds into the cerebrum, the remaining amounts of test and reference compounds in the ipsilateral cerebrum are measured. The reference compound, which does not cross the BBB, is used to determine the amount of test compound injected. Apparent efflux clearance reflects the efflux transport process across the BBB, but not across the BCSFB. The *in vivo *efflux clearance from CSF has been evaluated by means of the intracerebroventricular administration method [24]. After intracerebroventricular administration of a test compound, the remaining amount of test compound in the CSF is determined. The molecular mechanisms responsible for BBB and BCSFB transport have been investigated by using our established conditionally immortalized rat/mouse brain endothelial cell line (TR/TM-BBB) [25,26], rat choroid plexus epithelial cell line (TR-CSFB) [27] and freshly isolated choroid plexus. TR/TM-BBB cells and TR-CSFB cells retain the in vivo expressions and functions of several transporters and are a suitable in vitro model for the BBB [28] and BCSFB [5], respectively. A number of transporters expressed at the BBB and BCSFB have been identified as playing roles in nutrient supply and drug distribution to the brain and the CSF by using TR/TM-BBB cells and TR-CSFB cells [6].

## Transport of GCs precursor amino acids and neurotransmitters across the BBB

**Figure 2:**
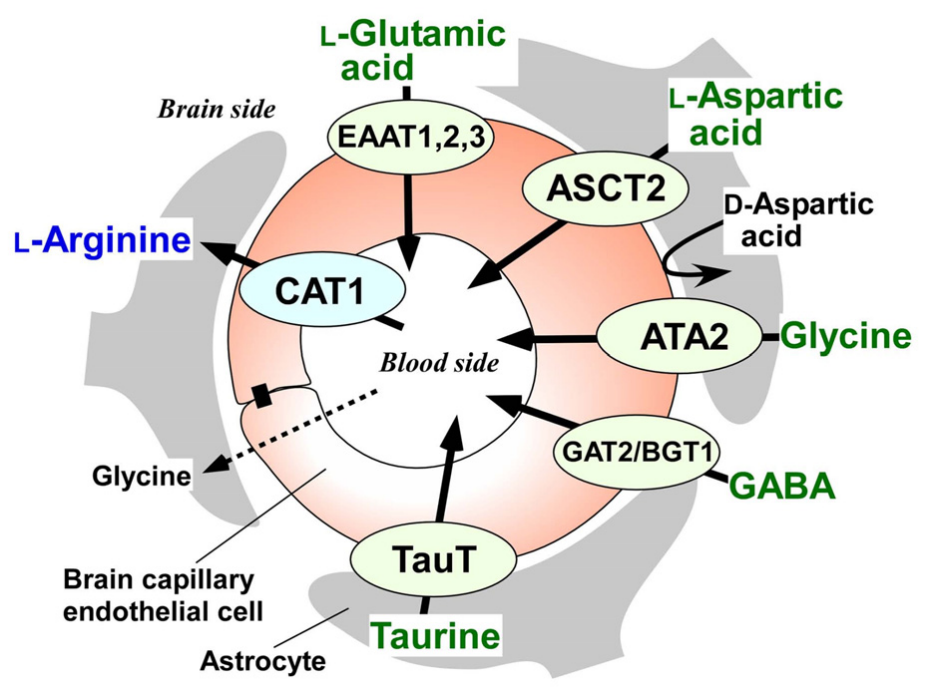
**Transport characteristics of guanidino compounds precursor amino acids and neurotransmitters at the BBB**.

The BBB possesses influx and efflux transport systems for GCs precursor amino acids and neurotransmitters, as illustrated in Figure 2. L-Arginine is transported from the circulating blood into the brain via Na^+^-independent cationic amino acid transporter 1 (CAT1/SLC7A1) expressed at the BBB [29,30]. The L-arginine influx transport at the rat BBB is saturable with a Michaelis-Menten constant (Km) value of 56 μM, which is lower than the physiological serum concentration of L-arginine in rat (170 μM [31]) and in human (100 μM [32]). Since L-arginine in mammals is derived mostly from renal de novo synthesis and dietary intake, CAT1 at the BBB would function as a supply pathway for L-arginine to the brain. The blood-to-brain transport of glycine across the BBB appears to be a passive diffusion, since [^14^C]glycine uptake by the brain was not inhibited by unlabeled glycine [20]. Zlokovic et al. [33] found, using the BUI method, that the BUI value of glycine was close to that of sucrose, used as a BBB impermeable paracellular marker. Since L-serine is possibly converted to glycine in the brain [34,35], glycine in the brain would be supplied by de novo synthesis, rather than via glycine influx transport at the BBB.

L-Glutamic acid, L-aspartic acid, glycine, γ-aminobutyric acid (GABA), and taurine undergo efflux from the brain into the circulating blood across the BBB [36-39]. A physiological role for the efflux transport processes at the BBB would be to remove the neurotransmitters and amino acids from the brain interstitial fluid and to maintain these extracellular concentrations below neurotoxic levels [40] if they had overflowed from neurons and glial cells under conditions of cerebral dysfunction, such as brain ischemia.

The brain-to-blood efflux transport across the BBB consists of two steps, i.e., influx across the abluminal membrane from the brain interstitial fluid into brain capillary endothelial cells and subsequent efflux across the luminal membrane from endothelial cells into the circulating blood. Na^+^-dependent transporters, such as EAAT (excitatory amino acid transporter) 1/SLC1A3, EAAT2/SLC1A2, and EAAT3/SLC1A1, for glutamate exist on the abluminal membranes of brain capillary endothelial cells [41]. These transporters mediate glutamate transport from the brain interstitial fluid into endothelial cells. When the glutamate concentration in endothelial cells becomes greater than that in the plasma, glutamate would be effluxed from the endothelial cells into the circulating blood in a facilitative manner [42,43]. Alanine-serine-cysteine transporter 2 (ASCT2/SLC1A5), mediates L-isomer-selective uptake of aspartic acid on the abluminal membrane of brain capillary endothelial cells in an Na^+^- and pH-dependent manner [44]. Na^+^-dependent amino acid transporter A2 (ATA2/SLC38A2) is predominantly expressed at the abluminal membrane of the brain capillary endothelial cells at the BBB and transports system A substrates, such as glycine and L-proline [39]. Mouse Na^+^-dependent GABA transporter 2/betaine-GABA transporter 1 (GAT2/BGT1/SLC6A12) is expressed in brain capillary endothelial cells and is involved in GABA efflux transport across the BBB [45]. Taurine efflux transport across the BBB is a saturable process with a Km of 39.1 μM [38]. This Km value is similar to that of rat taurine transporter (TauT/SLC6A6) [46], suggesting that TauT is involved in taurine efflux transport across the BBB.

In contrast to L-glutamic acid, L-aspartic acid, glycine, GABA, and taurine, not only is D-aspartic acid retained in the brain parenchymal cells, but also there is no efflux transport system for D-aspartic acid at the BBB [36]. This would facilitate the accumulation of D-aspartic acid in the brain. While the BBB acts as an efflux pump for L-aspartic acid and L-glutamic acid to reduce the brain interstitial concentration, it acts as a static wall for D-aspartic acid.

## Characteristics of transporters for GCs

Several transporters which belong to the SLC6 gene family are likely to accept GCs as substrates (Table 1). The SLC6 members are Na^+^- and Cl^-^-dependent transporters for neurotransmitters, amino acids and osmolytes, including GABA, betaine, taurine, and CT [47]. CRT mediates transport of CT, GAA, and CTN, with Km values of 46 μM/29 μM [48,49], 269 μM/412 μM [50], and 52 mM [51], respectively. The transport affinities of GAA and CTN for CRT are one and three orders of magnitude lower than that of CT, respectively. CT competitively inhibits CRT-mediated GAA transport with a Ki value of 60.5 μM [50]. Therefore, it is necessary to consider the physiological fluid concentrations of CT and GAA in order to evaluate the actual contribution of CRT to GAA transport. Since CRT is an electrogenic transporter, CT induces an inward current in *Xenopus *oocytes expressing human CRT at a holding potential of -60 mV [52]. GAA, GPA or GBA at the concentration of 1 mM induces significant inward current in CRT-expressing, but not water-injected, oocytes [50]. In the inhibition study, CRT-mediated GAA transport was significantly inhibited by GES, but not by taurine or GABA [50]. PCT and CTN induce inward current to a lesser extent in CRT-expressing oocytes, while GSA and MG do not induce inward current [50]. Thus, GPA, GBA and GES are potent substrates for CRT, whereas CRT does not recognize GSA, MG, taurine or GABA as a substrate. Dodd and Christie [53] have reported that two or three amino acid substitutions result in the loss of CT transporter activity and gain of a specific GABA transporter function. Because GPA inhibits mouse GABA transporter 3 and 4 (GAT3/SLC6A13 and GAT4/SLC6A11) [54,55], there is a possibility that mouse GAT3 and GAT4 mediate transport of GCs. Therefore, these amino acid residues may be involved in GAT-mediated transport of GCs which are not CRT substrates. TauT accepts taurine, GAA, and GABA as substrates with Km values of 43 μM [46], 215 μM [56] and 1.46 mM [57], respectively. The transport affinities of GAA and GABA for TauT are approximately one and two orders of magnitude lower than that of taurine, respectively. TauT-mediated GAA uptake is significantly inhibited by taurine, GABA, GPA, GBA and GES, whereas CT, GSA, MG and CTN have no effect [56]. This result suggests that GPA, GBA and GES are good substrates for TauT, whereas CT, GSA, MG, and CTN are not.

**Table 1:** Inhibitory effect of various compounds on [^14^C]GAA uptake by HEK293 cells stably overexpressing CRT (CRT/HEK293 cells) and *Xenopus *oocytes expressing CRT (CRT/oocytes) or TauT (TauT/oocytes), and [^14^C]CTN uptake by *Xenopus *oocytes expressing rOCT3(rOCT3/oocytes).

Inhibitor		[^14^C]GAA uptake		[^14^C]CTN uptake
	
	CRT/HEK293 cells %of control	CRT/oocytes %of control	TauT/oocytes %of control	rOCT3/oocytes %of control
Control	100 ± 4	100 ± 9	100 ± 9	100 ± 19
Guanidinoacetate (GAA)	19.2 ± 2.6*	44.6 ± 2.7*	53.2 ± 9.6*	-
β-Guanidinopropionate (GPA)	1.81 ± 0.14*	0.944 ± 0.189*	4.77 ± 0.66*	143 ± 21
γ-Guanidinobutyrate (GBA)	10.0 ± 0.6*	21.4 ± 2.0*	21.7 ± 4.2*	-
Guanidinoethansulfonate (GES)	23.1 ± 1.1*	-	4.66 ± 0.22*	-
Guanidinosuccinate (GSA)	101 ± 1	107 ± 12	103 ± 10	-
Creatine (CT)	3.06 ± 0.24*	2.91 ± 0.23*	98.4 ± 12.7	137 ± 24
Taurine	111 ± 5	-	3.00 ± 0.19*	-
γ-Aminobutyric acid (GABA)	95.5 ± 2.9	-	34.0 ± 4.6*	-
Creatinine (CTN)	93.3 ± 5.3	123 ± 10	97.9 ± 14.6	-
Methylguanidine (MG)	82.3 ± 15.2	126 ± 14	110 ± 18	47.2 ± 18.9**
Creatine phosphate (PCT)	93.4 ± 6.8	97.9 ± 14.1	117 ± 6	-
Guanidine	114 ± 6	88.2 ± 13.4	-	137 ± 22
L-Arginine	86.6 ± 6.3	-	-	-
Glycine	126 ± 4	-	-	-
L-Alanine	111 ± 3	-	-	-
L-Aspartic acid	98.5 ± 2.2	-	-	-
Tetraethylammonium	128 ± 29	82.8 ± 7.3	-	48.1 ± 8.3**
Benzylpenicillin	110 ± 14	115 ± 12	-	-

[^14^C]GAA (20-45 μM) and [^14^C]CTN (70 μM) uptake by CRT/oocytes, TauT/oocytes and rOCT3/oocytes was measured at 20°C for 60 min in the absence (control) or presence of inhibitors (2 mM). [^14^C]GAA (3 μM) uptake by CRT/HEK293 cells was measured at 37°C for 5 min in the absence (control) or presence of inhibitors (2 mM). Each value represents the mean ± SEM (n = 4-8 for CRT/HEK293 cells, n = 10-25 for CRT/oocytes, TauT/oocytes and rOCT3/oocytes). *p < 0.01, **p < 0.05, significantly different from control. -, not determined.

Modified and reproduced from Tachikawa et al. [50,51,56].

Rat organic cation transporter 3 (rOCT3/SLC22A3) and human OCT2 (hOCT2/SLC22A2) mediate low-affinity CTN transport with Km values of 47.7 mM [51] and 4.0 mM [58], respectively. In contrast, human OCT1 (hOCT1/SLC22A1) does not recognize CTN [58]. MG inhibits rOCT3-mediated CTN transport [51], implying that MG is also a substrate for rOCT3. Guanidine, GSA and MG inhibit hOCT1 and/or hOCT2-mediated tetraethylammonium uptake, while GAA has little effect on the uptake [59]. Thus, the SLC22 members are likely to mediate transport of GSA, CTN, guanidine, and MG, which are not recognized by the SLC6 family members.

## Transport of CT across the BBB

In human brain, the CT concentration (7.4 mM) is approximately 190-fold greater than that in the plasma (39.5 μM) [3]. *In vivo *analysis has revealed that CT is transported from the circulating blood to the brain via a carrier-mediated transport system at the BBB [21]. The [^14^C]CT influx transport clearance across the BBB was 1.61 μL/(min•g brain), which is about 6-fold greater than that of [^14^C]sucrose [60], a BBB impermeable paracellular marker in rats. The apparent cerebrum-to-plasma concentration ratio of [^14^C]CT reaches 30.8 mL/g tissue 24 hours after exogenous administration of [^14^C]CT to mice. This value is consistent with the endogenous brain-to-serum concentration ratio of CT [3], suggesting that the BBB functions as a major pathway for supplying CT to the brain from the circulating blood.

The characteristics of [^14^C]CT uptake by TM-BBB cells support the idea that CRT is involved in CT transport at the BBB [21]. [^14^C]CT uptake by TM-BBB cells takes place in an Na^+^-, Cl^-^- and concentration-dependent manner with a Km value of 16.2 μM, which is consistent with the apparent Km values of 29 μM for human CRT [61] and 15 μM for rat CRT [48]. GPA causes marked inhibition of [^14^C]CT uptake by TM-BBB cells as reported elsewhere [62]. The corresponding Km value is 10- to 40-fold lower than the plasma concentration (140-600 μM) in mouse and rat [2]. The blood-to-brain transport of CT is more than 90% saturated by endogenous plasma CT, and CRT at the BBB plays a role in continuously supplying CT from the circulating blood to the brain at a constant rate equivalent to the maximal velocity. This suggests that the efficacy of oral CT treatment for patients with neurodegenerative diseases may depend on the CRT function at the BBB. Belanger et al. [63] have found that the mRNA expression and function of CRT in TM-BBB cells are increased under hyperammonemic conditions. CRT function at the BBB might be highly regulated under pathophysiological conditions.

Immunohistochemical studies have indicated that CRT is localized at both the luminal and abluminal sides of mouse brain capillary endothelial cells in the adult brain [21,64]. CRT is also expressed in neurons [21,65], suggesting that CT is transported into neurons by CRT following BBB transport. In support of this notion, *in vitro *studies reveal that an exogenous supply of CT increases the neuronal PCT store and protects neurons from hypoxic damage, glutamate excitotoxicity and β-amyloid-induced toxicity [66,67]. Oral administration of CT has been reported to protect neurons in animal models of amyotrophic lateral sclerosis, Huntington's disease and Parkinson's disease [68-70]. Thus, the processes of CT transport at the BBB are important for understanding the mechanism governing the supply of CT to the brain and information about them could help in the design of improved oral CT supplementation for the treatment of neurodegenerative diseases and CDS.

## Transport of GAA across the BBB and BCSFB

GAA is present at low concentrations (2-12 μM) in normal mammalian brain [3]. In contrast, the GAA level in the brain of GAMT-knockout mice (1.85-1.87 mM) is approximately 150-fold greater than that in normal brain (12 μM) [71,72]. Since GAA exerts an epileptogenic effect by affecting GABAergic neurotransmission [73], an increased level of GAA in the brain appears to affect brain function. We have investigated why GAA is abnormally accumulated in the brains of patients with GAMT deficiency.

### Brain-to-blood efflux transport of GAA across the BBB and BCSB

*In vivo *and *ex vivo *studies reveal that there is no efflux transport of GAA across the BBB, and instead GAA undergoes efflux transport from the CSF [50,56]. [^14^C]GAA microinjected into rat cerebrum was not effluxed from the brain across the BBB [56], whereas the elimination clearance of GAA via the BCSFB (3.97 μL/min per rat) [50] is still approximately 33-fold greater than the apparent blood-to-CSF influx clearance of GAA (0.122 μL/min per rat) [56]. This predominant GAA efflux transport at the BCSFB can explain the fact that the GAA concentration in human CSF (0.036-0.22 μM) is almost one order of magnitude lower than that in plasma (0.35-3.5 μM) [8]. This also may help to explain the rapid removal of increased GAA from the CSF at the onset of convulsions [19], preventing the continuation of convulsions and seizures.

GAA transport at the BCSFB is mediated at least partially by CRT and TauT, which may make approximately 50% and 11% contributions to total GAA uptake by the choroid plexus, respectively [50,56]. The functional expression of CRT at the BCSFB is supported by the brush-border membrane localization of CRT in choroid plexus epithelial cells and the observation of Na^+^- and Cl^-^-dependent [^14^C]CT uptake by TR-CSFB [50]. TauT appears to be expressed on the brush-border membrane of choroid plexus epithelial cells [74]. The Km value of Na^+^-dependent GAA uptake by TR-CSFB cells (929 μM) is comparable with that of GAA uptake by CRT-expressing HEK293 cells (412 μM)/*Xenopus laevis *oocytes (269 μM), as well as that of TauT-expressing *Xenopus laevis *oocytes (215 μM) [50,56]. The *in vivo *process of GAA elimination from the CSF was partially inhibited by CT and taurine [50,56].

Interestingly, one clinical report has demonstrated that patients with CRT deficiency exhibit increased GAA levels, as well as the absence of CT in the brain [75], implying a causal relationship between the gene defect of CRT and the increase in brain GAA level. Although neurological complications in patients with CRT deficiency would be caused mainly by the lack of CT transport in neuronal cells, the dysfunction of CRT may also lead to an increase in GAA in the brain, owing to the lack of CRT-mediated GAA efflux transport across the BCSFB. In patients with GAMT deficiency, GAA is accumulated in the CSF (11-15 μM), whereas CT levels in the CSF are significantly decreased (< 2.0 μM). However, GAA concentration in the CSF of these patients is still lower than that in the brain parenchyma [76] or plasma [8]. This is because CRT and TauT would play an important role in the efflux transport of GAA from the CSF at the BCSFB. Indeed, the Km value of GAA uptake by TR-CSFB cells (926 μM) [50] is greater than the GAA concentration in the CSF of the patients (11-15 μM [76]).

### Blood-to-brain influx transport of GAA across the BBB

GAA influx transport via CRT at the BBB would also play a key role in the cerebral accumulation of GAA in patients with GAMT deficiency. The characteristics of Na^+^- and Cl^-^-dependent [^14^C]GAA uptake by TR-BBB cells are consistent with those of CRT, but not TauT [50]. The Km value of 556 μM in TR-BBB cells is comparable with that obtained for GAA uptake using CRT-expressing *Xenopus laevis *oocytes (269 μM)/HEK293 cells (412 μM). Taurine has no significant effect on the GAA uptake. CRT is localized in mouse brain capillary endothelial cells and mediates CT influx transport across the BBB [21,64]. Considering that the Km value of rat CRT-mediated CT transport is 46 μM/29 μM [48,49], the function of CRT on the luminal membrane could be 85-90% saturated by endogenous CT in the serum under physiological conditions (250 μM in rats [3]). Thus, it appears that CRT-mediated transport of GAA at the BBB is almost wholly inhibited by CT in the blood under normal conditions. In patients with GAMT deficiency, the CT levels in the plasma (1-5 μM) are smaller than reference values (6-109 μM), whereas the GAA levels in the plasma (12-39 μM) are greater than reference values (0.35-3.5 μM) [8]. Although the influx and efflux transport of GAA across the brain barriers are well balanced, at least under normal conditions [56], the degree of CRT saturation by endogenous CT in the circulating blood of the patients would be smaller, thus contributing to the significant increase of the blood-to-brain GAA transport. This mechanism could explain the fact that treatment with CT supplementation resulted in partial normalization of cerebral levels of CT and GAA in patients [77].

### GAA transport in brain parenchymal cells

[^14^C]GAA is taken up by brain parenchymal cells in a concentrative manner, presumably via CRT, TauT and other Na^+^-dependent carrier-mediated system(s) [56]. This would be another reason for the accumulation of GAA in the brain of patient with GAMT deficiency. The contribution of CRT to the GAA uptake by brain parenchymal cells appears to be small, because CT does not inhibit the GAA uptake by more than 11% even at a concentration of 10 mM. It has been reported that there is a relationship between gene polymorphism of CRT, transport activity of CRT, and neurological symptoms such as developmental delay, mental disorder and epilepsy with seizures [78-80]. Almeida et al. [81] proposed that exocytotic release of CT into the brain interstitial fluid acts as a neuromodulator of GABAergic neurotransmission. Thus, CRT may be mainly responsible for the uptake of CT by CRT-expressing neurons and oligodendrocytes to maintain the proper GABAergic neurotransmission. GPA inhibits the GAA uptake by brain slices by 89%, which is greater than the inhibitory effect of taurine plus CT (59%), but similar to the effect of Na^+^-depletion (92%) [56]. Since GPA is a potent inhibitor of mouse GAT3 and GAT4 [54,55], as well as of CRT- and TauT-mediated GAA uptake (Table 1), there is a possibility that the GATs play a role in GAA uptake.

In summary, the key factors facilitating GAA accumulation in the brains of patients with GAMT deficiency could be (i) the lack of efflux transport across the BBB, (ii) the increase of CRT-mediated blood-to-brain GAA transport at the BBB, and (iii) the Na^+^-dependent concentrative GAA transport by brain parenchymal cells.

## Transport of CTN across the BBB and BCSFB

Several GCs, such as CTN, MG, guanidine, and GSA, are involved in the typical manifestations, such as epileptic and cognitive symptoms, of uremic encephalopathy [82]. Levels of uremic GCs are substantially increased in serum, CSF, and brain of nondialyzed patients with renal insufficiency [83,84]. The highest CTN concentration in the CSF of severely uremic patients is 521 μM, which is about 8-fold greater than that in control subjects (67.6 μM) [82]. The uremic GC concentrations observed in the brain of uremic patients are similar to those producing convulsive effects [82], and so could contribute to the neurological complications suffered by these patients [85]. Since no specific saturable uptake mechanism exists for CTN in human red blood cells [86], it has been postulated that CTN diffuses out of the brain. In the kidney, CTN is excreted into urine by tubular secretion, as well as glomerular filtration. Human OCT2, but not hOCT1, mediates the tubular secretion of CTN at the basolateral membrane of renal proximal tubular cells [58]. These findings pose a question as to whether CTN undergoes efflux from the brain only by diffusion.

### Brain-to-blood efflux transport of CTN across the BBB and BCSFB

Emerging evidence indicates that the BCSFB is the major pathway of cerebral CTN clearance, and transporter-mediated processes, at least in part via rOCT3, are involved in CTN efflux transport from the CSF at the BCSFB [51]. CTN, after intracerebral administration, is not significantly eliminated from the brain across the BBB, whereas the elimination clearance of CTN from the CSF is 60-fold greater than that of inulin (reflecting CSF bulk flow). Even in renal failure model rats, the ratio of increase of the CTN concentration in CSF was smaller than that in plasma, suggesting a significant role for the CSF-to-blood efflux process. The inhibitory effects of inhibitors and antisense oligonucleotides on [^14^C]CTN uptake by isolated choroid plexus indicate the involvement of rOCT3 and CRT in CTN transport. Rat OCT3 is functionally expressed in choroid plexus epithelial cells [87]. Since endogenous compounds in the CSF may affect rOCT3- and CRT-mediated transport of CTN in the choroid plexus, the physiological conditions in the CSF should be taken into account in evaluating the actual contributions of rOCT3 and CRT. Considering the Km value of CRT for CT (46 μM/29 μM [48,49]) and the normal CSF concentration of CT (24-66 μM [8]), CRT on the brush-border membrane of the choroid plexus epithelial cells may be approximately 50% saturated by endogenous CT in the CSF. Furthermore, the Km value of CRT for CTN (52 mM) [51] is almost 3 orders of magnitude greater than the Km value of CRT for CT. Thus, it is unlikely that CRT plays a role in the elimination of CTN from the CSF. Therefore, it appears that CTN efflux transport, at least in part via rOCT3, at the BCSFB plays a crucial role in the efficient removal of CTN from the CSF. Indeed, the Km value of rOCT3-mediated CTN transport (47.7 mM) [51] is still much higher than the CTN concentration in the CSF of patients with renal failure (168-521 μM [85]), suggesting that rOCT3 functions as a CTN transporter without saturation.

### Blood-to-brain influx transport of CTN across the BBB

The blood-to-brain influx clearance of CTN is approximately 14-fold greater than that of sucrose, used as a BBB impermeable paracellular marker [60]. This evidence implies that the blood-to-brain transport of CTN is carrier-mediated, rather than occurring simply by passive diffusion. In this regard, elevated levels of CTN in the brain of the patients with renal insufficiency might be due to increased permeability of CTN in the blood-to-brain direction. Therefore, it will be clinically beneficial, as far as the cerebral accumulation of CTN is concerned, to block the BBB influx transport of CTN in patients with renal insufficiency.

## Conclusion

**Figure 3:**
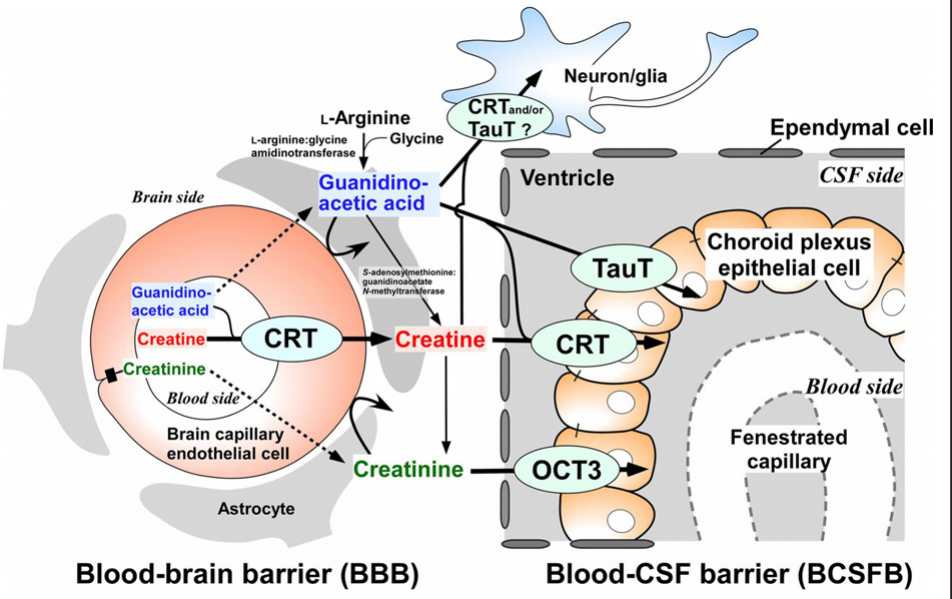
**Transport characteristics of guanidino compounds at the BBB and the BSCFB**.

Our recent research provides a novel insight into how physiologically important and/or neurotoxic GCs are accumulated in the brain under physiological and pathological conditions. Transporters for GCs at the BBB and BCSFB have emerged as determinants of GCs distribution to the brain as summarized in Figure 3. CRT expressed at the BBB regulates the CT concentration in the brain at millimolar levels as a major pathway for supplying CT from the circulating blood to the brain. CRT may also be a key factor in blood-to-brain GAA transport in patients with GAMT deficiency, causing abnormal accumulation of GAA in the brain. CRT, TauT and OCT3 expressed at the BCSFB are involved in GAA and/or CTN efflux transport from the CSF. Interestingly, BBB efflux transport of GCs, including GAA and CTN, is negligible, though the BBB has a variety of efflux transport systems for synthetic precursors of GCs, such as amino acids and neurotransmitters. Instead, the BCSFB functions as a major cerebral clearance system for GCs. These findings should help us identify the molecular mechanism(s) of neurological complications in unexplained convulsions and seizures. The regulatory mechanisms of GCs transport at the BBB and BCSFB need to be further clarified to provide a more rational basis for therapy of neuronal disorders caused by accumulation of GCs and insufficiency of CT in the brain. Such information should also aid the discovery and development of drugs which facilitate efflux transport of GCs at the BBB and the BCSFB in order to prevent accumulation of GCs, as well as drugs which facilitate the influx transport of CT at the BBB to promote efficient supplementation of CT.

## Abbreviations

AGAT: L-arginine:glycine amidinotransferase; ASCT: alanine-serine-cysteine transporter; ATA: amino acid transporter A; BBB: blood-brain barrier; BCSFB: blood-cerebrospinal fluid barrier; BEI: brain efflux index; BGT: betaine- γ-aminobutyric acid transporter; BUI: brain uptake index; CAT: cationic amino acid transporter; CDS: creatine deficiency syndrome; CRT: creatine transporter; CT: creatine; CTN: creatinine; CSF: cerebrospinal fluid; EAAT: excitatory amino acid transporter; GAA: guanidinoacetic acid; GABA: γ-aminobutyric acid; GAMT: *S*-adenosylmethionine:guanidinoacetate *N*-methyltransferase; GAT: γ-aminobutyric acid transporter; GBA: γ-guanidinobutyric acid; GC: guanidino compound; GES: guanidinoethane sulfonic acid; GGA: α-guanidinoglutaric acid; GPA: β-guanidinopropionic acid; GSA: guanidinosuccinic acid; MG: methylguanidine; OCT: organic cation transporter; PCT: phosphocreatine; SLC: solute carrier; TauT: taurine transporter;

## Competing interests

The authors declare that they have no competing interests.

## Authors' contributions

All authors contributed to the writing of this review. All authors have read and approved the final version of the manuscript.
